# Visual field improvement after endoscopic transsphenoidal surgery in patients with pituitary adenoma

**DOI:** 10.3389/fonc.2023.1108883

**Published:** 2023-02-15

**Authors:** Xiaoyu Ji, Xinyu Zhuang, Siyuan Yang, Kai Zhang, Xiaozhe Li, Kun Yuan, Xiaofeng Zhang, Xuebo Sun

**Affiliations:** ^1^ Department of Neurosurgery, The First Affiliated Hospital of Soochow University, Suzhou, China; ^2^ Department of Ophthalmology, The First Affiliated Hospital of Soochow University, Suzhou, China; ^3^ Department of Ophthalmology, Dushu Lake Hospital Affiliated to Soochow University, Suzhou, China

**Keywords:** pituitary adenoma, visual field, nomogram, endoscopic transsphenoidal surgery, improvement

## Abstract

**Purpose:**

To analyze and predict the possibility of visual field (VF) recovery after endoscopic transsphenoidal surgery (ETSS) in patients with pituitary adenoma, we investigated the factors affecting the improvement of the visual field defect (VFD) and built a nomogram predictive model based on these risk factors. We further investigated specific recovery regions of VF associated with the improvement of VFD.

**Methods:**

The clinical data of patients who underwent ETSS for pituitary adenomas at a single center between the January 2021 and April 2022 were retrospectively analyzed. Univariate and multivariate analyses were used to determine the predictive factors affecting the improvement in the VF defect and specific recovery regions in patients with pituitary adenomas after ETSS.

**Results:**

We enrolled 28 patients (56 eyes) who were hospitalized at our institution. Four clinical features, including compression of the optic chiasm, preoperative mean defect (MD), diffuse defect, and duration of the visual symptom, were chosen from the least absolute shrinkage and selection operator regression analysis to establish the predictive nomogram. The nomogram’s area under the curve (AUC) was 0.912, indicating a good degree of differentiation. A calibration plot was used to evaluate the predictive model’s calibration, and a decision curve was used to evaluate its clinical application value. The VF defects were improved in the 270–300° range (270–300: RR = 361.00, 95% CI: 21.01–6,202.41).

**Conclusion:**

We developed a predictive nomogram model based on significant visual field improvement-associated factors after ETSS in patients with pituitary adenoma. Postoperative visual field improvement is likely to begin at 270–300° in the inferior temporal quadrant. This improvement would enable personalized counselling for individual patients by precisely predicting the visual field recovery after surgery.

## 1 Introduction

Pituitary tumors account for 10-15% of all diagnosed primary intracranial lesions (1), and in 30-70% of cases, they cause chiasmal compression and associated visual impairments (2, 3). Visual symptoms result from compression and ischemia of the optic chiasm. Visual field loss is the main symptom, `particularly bitemporal hemianopia (4). The progression of a visual field defect (VFD) changes from superior to inferior in the temporal hemifield and from inferior to superior in the nasal hemifield (5). VFD and decreased visual acuity are important indications for transsphenoidal surgery for pituitary adenomas (6–9). A recent meta-analysis found that VFD improved after endoscopic endonasal transsphenoidal surgery (ETSS) in most patients with pituitary tumors, with a prevalence rate of improvement of nearly 80.8% (10).The microscopic transcranial approach is a useful strategy for about 1-10% of large-to-giant pituitary adenomas. However, the risk of postoperative visual outcomes worsening could be as high as 25%. Subarachnoid space invasion identified by preoperative magnetic resonance imaging was the only independent negative predictor for visual function after transcranial surgery (11).

Although ETSS is an effective and safe treatment for patients with VFD, complete recovery from VFD is rare. Existing studies of visual field (VF) recovery mainly analyzed the overall VF map and lacked results for specific defective regions.

In addition, previous studies have focused on the factors influencing postoperative visual field recovery, including the duration of symptoms, the preoperative mean defect (MD), age, retinal nerve fiber layer (RNFL) thickness, tumor volume, suprasellar tumor extension, and expression levels of vascular endothelial growth factor (VEGF)/Ki-67 (12–15).

To analyze and predict the possibility of VF recovery after ETSS in patients with pituitary adenoma, we investigated the factors affecting the improvement of VFD and developed a dynamic nomogram predictive model based on these risk factors. We also studied the specific regions of VF recovery associated with the improvement of VFD.

## 2 Materials and methods

### 2.1 Patient population and study design

Patients with pituitary adenomas were identified by histopathology and magnetic resonance imaging (MRI) at the First Affiliated Hospital of Soochow University between January 2021 and April 2022. All participants underwent pre- and postoperative ophthalmic examinations, including visual acuity and visual field measurements. The visual field map was divided into the superior temporal region (0–90°), inferior temporal region (270–360°), superior nasal region (90–180°), and inferior nasal regions (180–270°). Each region was divided into three ranges (0–30°, 30–60°, 60–90°, 90–120°, 120–150°, 150–180°, 180–210°, 210–240°, 240–270°, 270–300°, 300–330°, and 330–360°).

The inclusion criteria were as follows: (1) pituitary adenomas identified by histopathological examination and MRI; (2) evidence of preoperative VF impairment; (3) endoscopic endonasal surgery; and (4) totally resection identified by postoperative MRI.

Exclusion criteria were as follows: (1) patients without defects in the preoperative visual field; (2) unavailable data or insufficient ophthalmic examinations; (3) previous treatment for ocular lesions and previous ocular surgery; (4) high myopia (>6 diopters); (5) congenital eye disease; (6) glaucoma, any optic disc anomaly, and macular disease.

### 2.2 Visual field testing

Static central visual field tests were performed using the Octopus 900 perimeter (Haag-Streit Inc., Koeniz, Switzerland). Standard automated perimetry was performed using tendency-oriented perimetry (G-TOP) strategy, which examined 59 testing points within 30° of the central visual field using Goldmann III stimuli with a stimulus duration of 100 ms under a background luminance of 31.4 asb (10/cd/m^2^). The tests were performed under low-light conditions. During the testing session, the better eye was tested first, and the non-examined eye of the patient was covered by an occluded white eye. Seven-in-one reports for each eye were collected, and MD was recorded before and after the operation, which is a promising way to measure quantitative outcomes (16).Healthy eyes were defined as those with an MD less than 2 dB. And visual field defect was defined as an Octopus perimetry MD higher than 2 dB. The outcome of interest was the improvement in VFD after ETSS. The diagnosis of improvement of VFD was defined based on MD difference values higher than 1 dB. The diffuse defect is defined as the average of the difference between the 20 and 27 percentile values of the cumulative defect curve in relation to the 50 percentile value (17).

### 2.3 Data collection

We collected the following data on patients who underwent ETSS for pituitary adenomas: patient basic information (age, sex, duration of visual symptoms); tumor characteristics (tumor maximum diameter identified by MRI, tumor texture, categorized as not hard (easily or difficultily removed by suction), or hard (not removable by suction and excised en bloc)) (18); radiological examination (optic chiasm compression identified *via* MRI); visual field testing (diffuse defect or not, preoperative MD); laboratory values on admission (Ki67, CD34, EGFR, Mmp9, P53, neutrophil (×10^9^/L), platelet (×10^9^/L), lymphocyte (×10^9^/L), neutrophil-to-lymphocyte ratio (NLR), systemic immune-inflammation index(SII)(×10^9^/L), and platelet-to-lymphocyte ratio (PLR)); and outcome (the improvement of VFD after ETSS).

### 2.4 Statistical analyses

All data were statistically analyzed using the R software version 4.2.0 (R Foundation for Statistical Computing, Vienna, Austria). Frequency (percentages) was used for categorical variables, while mean ± standard deviation and median (interquartile range) were used for continuous variables. Nominal data were compared using the Chi-squared or Fisher’s exact test. For comparisons among these groups, ANOVA and Mann-Whitney U-tests were performed depending on the mode of data distribution.

The least absolute shrinkage and selection operator (LASSO) regression method is a shrinkage method that can actively select from a large and potentially multicollinear set of variables in the regression and can result in a more relevant and interpretable set of predictors (19). This method can reduce the size of the coefficients of the independent variables according to their predictive power. Variables were selected to identify the optimum combination of variables that could predict the improvement of VFD in patients with pituitary tumors using the LASSO regression model. During the LASSO analysis, the built-in function in R produces two automatic λ’s: one that minimizes the binomial deviance and one representing the largest λ that is still within one standard error of the minimum binomial deviance. We opted for the largest λ as it results in a stricter penalty, allowing us to reduce the number of covariates even further than the deviance-minimizing λ.

Finally, after selecting the optimal combination of variables, a dynamic nomogram (https://cerebralnomogram.shinyapps.io/Pituitary_adenoma_IVFDnom/) was created. Next, the discriminative ability of the nomogram was summarized using the receiver operating characteristic curve. The uniformity between the nomogram and the ideal observation was evaluated *via* the calibration curve, and the calibration plots on the slope of the 45° line were considered excellent models. A decision curve was used to evaluate the clinical application of the nomogram. To research the differences of the difficulty in the improvement of visual field defect among the divided twelve ranges. Thus, we compare the improvement rate of visual field defect in each ranges (dichotomous variables) using the Mantel-Haenszel method *via* Revman software. A P>0.05 is defined as significance difference.

## 3 Results

### 3.1 Baseline characteristics of the patients included

**Figure 1:**
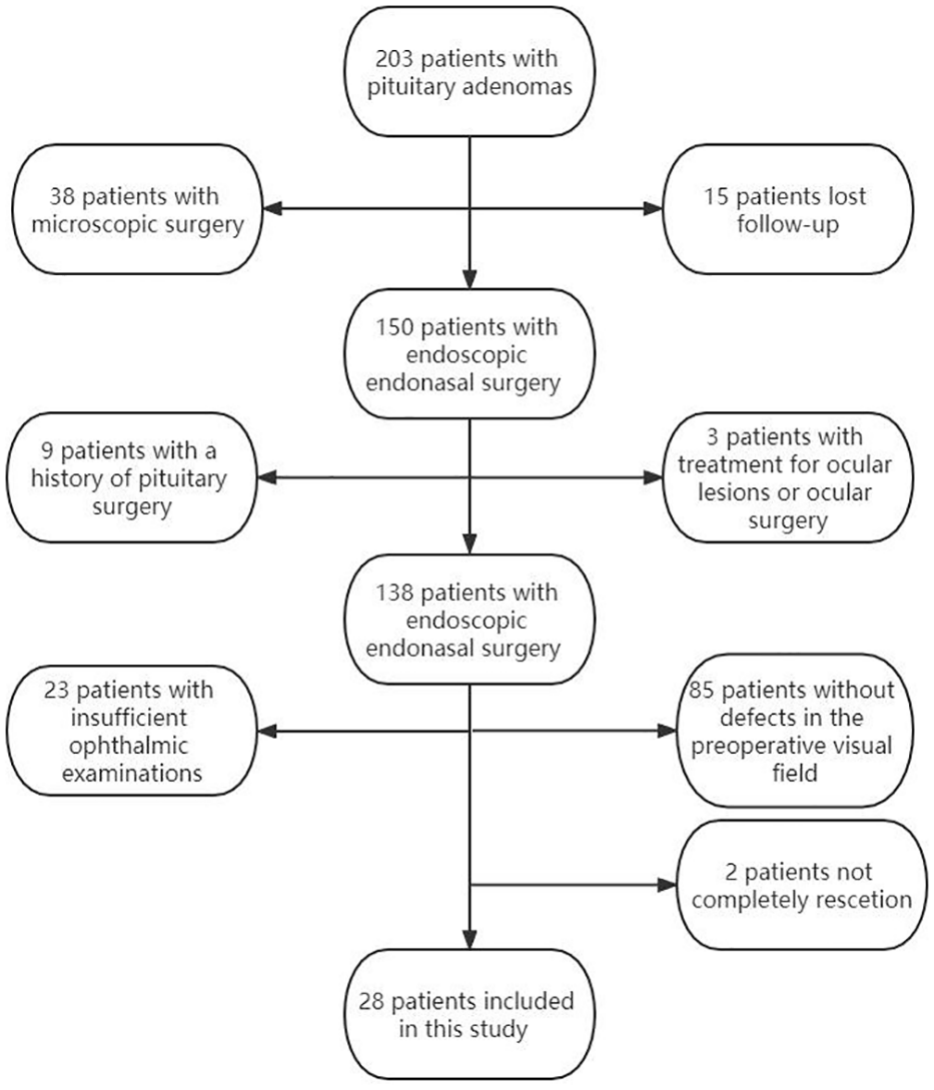
Flowchart of study participants.

The study finally enrolled a 28 patients (56 eyes) according to the inclusion and exclusion criteria. (**Figure 1**). The characteristics of the patients with pituitary tumors and patient's eyes included in our study are summarized in **Tables 1**, **2**.

**Table 1:** Overview of patient characteristics.

Category (units)	Overall patients
No. of patients	28
Age, mean(SD)	45.1 (14.7)
Male sex, n%	10 (35.71%)
Symptoms	
diplopia	13 (46.43%)
headache	5 (17.86%)
irregular menstruation	5 (17.86%)
dizziness	3 (10.7%)
decreased sexual potency	2 (7.14%)
acral enlargement	2 (7.14%)
facial acne	2 (7.14%)

**Table 2:** Baseline characteristics of eyes of patients included.

Variables	Overall eyes	Unimproved	Improved	*P*-value
Patients, n (%)	56	28	28	
Male, n (%)	20 (35.7)	10 (35.7)	10 (35.7)	1.000
Age in years, median (IQR)	41.50(35.25, 53.25)	41.50 (37.50, 51.00)	44.50 (30.25, 62.00)	0.793
Symptom duration before surgery (months), median [IQR]	2.50 (0.00, 12.00)	10.50 (0.00, 24.00)	1.00 (0.00, 5.25)	0.042
Optic nerve compression, n (%)	36 (64.3)	13 (46.4)	23 (82.1)	0.012
Tumor maximum diameter, median [IQR]	20.00 (15.00, 29.25)	20.00 (14.50, 21.00)	25.00 (19.25, 33.50)	0.011
Hard tumor texture, n (%)	26 (46.4)	13 (46.4)	13 (46.4)	1.000
Diffuse defect, n (%)	30 (53.6)	9 (32.1)	21 (75.0)	0.003
Pre-operation MD, mean (SD)	7.39 (6.03)	5.27 (5.67)	9.51 (5.70)	0.007
Immunohistochemistry (IHC)				
Ki-67, median [IQR]	3.00 (2.00, 4.25)	3.00 (3.00, 5.00)	3.00 (2.00, 4.00)	0.271
CD34, n (%)	48 (85.7)	26 (92.9)	22(78.6)	0.252
EGFR, n (%)	18 (32.1)	13 (46.4)	5 (17.9)	0.045
Mmp9, n (%)	16 (28.6)	10 (35.7)	6 (21.4)	0.375
P53, n (%)	46 (82.1)	24 (85.7)	22 (78.6)	0.727
Admission Laboratory Values				
Neutrophil (×10^9^/L), median (IQR)	3.55 (3.06, 4.06)	3.56 (2.97, 4.10)	3.55 (3.12, 3.95)	0.974
Lymphocyte (×10^9^/L), median (IQR)	1.91 (1.55, 2.40)	1.81 (1.57, 2.36)	2.10 (1.51, 2.41)	0.533
Platelet (×10^9^/L), median (IQR)	217.00 (187.75, 251.75)	218.00 (180.00, 257.50)	214.50 (197.50, 241.00)	0.844
NLR, median (IQR)	1.78 (1.32, 2.42)	1.80 (1.32, 2.43)	1.75 (1.31, 2.18)	0.555
PLR, median (IQR)	111.65 (97.57, 142.30)	113.67 (100.35, 155.83)	107.61 (87.63, 132.06)	0.325
SII index (×10^9^/L), median (IQR)	380.53 (316.18, 594.15)	430.61 (316.80, 625.87)	361.50 (314.30, 447.26)	0.325

### 3.2 LASSO regression analysis of factors associated with the improvement of VFD in patients with a pituitary tumor

**Figure 2:**
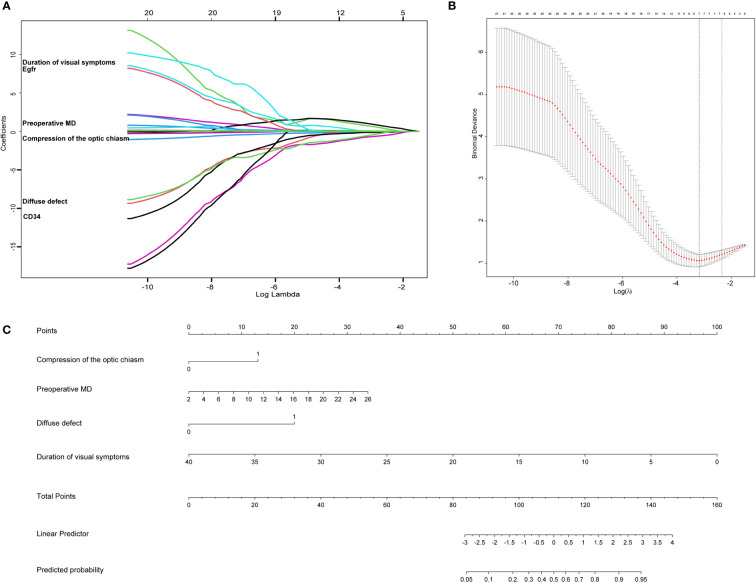
The lasso analysis and nomogram for predicting the improvement of visual field defect in patients with pituitary tumor. **(A)** LASSO model selecting most useful parameters. **(B)** LASSO coefficient graph of the key parameters. **(C)** The nomogram including compression of the optic chiasm, preoperative MD, diffuse defect, duration of visual symptoms.

Univariate Logistic regression analysis for improvement of visual field defect of patients with pituitary tumor are summarized in **Table 3**. LASSO regression was applied to filter the 21 variables across 56 eyes of 28 patients with a pituitary tumor. According to the λ that is still within one standard error of the minimum binomial deviance, four variables (compression of the optic chiasm, preoperative MD, diffuse defect, and duration of visual symptoms) were selected for further stepwise analysis to make up the optimum model (**Figures 2A, B**).

**Table 3:** Univariate logistic regression analysis for improvement of visual field defect of patients with pituitary tumor.

	Improvement of visual field defect		
	Unadjusted		
Variables	SE	OR (95% CI)	*P*-value
Male	5.578e-01	1.00 (0.33, 3.01)	>0.9
Age in years	0.019	1.01 (0.98, 1.05)	0.542
Symptom duration before surgery (months)	0.044	0.88 (0.80, 0.95)	0.005
Optic nerve compression	0.622	5.31 (1.65, 19.5)	0.007
Tumor maximum diameter	0.030	1.07 (1.01, 1.14)	0.028
Hard tumor texture	5.359e-01	1.00 (0.35, 2.88)	>0.9
Diffuse defect	0.595	6.33 (2.06, 21.6)	0.002
Pre-operation MD	0.056	1.15 (1.04, 1.30)	0.013
Immunohistochemistry(IHC)			
Ki-67	0.128	0.90 (0.69, 1.15)	0.387
CD34	0.866	0.28 (0.04, 1.37)	0.144
EGFR	0.622	0.25 (0.07, 0,81)	0.026
Mmp9	0.606	0.49 (0.14, 1.58)	0.241
P53	0.710	0.61 (0.14, 2.42)	0.488
Admission Laboratory Values			
Neutrophil (×10^9^/L)	0.148	0.97 (0.72, 1.30)	0.833
Lymphocyte (×10^9^/L)	0.394	1.67 (0.80, 3.87)	0.194
Platelet (×10^9^/L)	5.180e-03	1.00 (0.99, 1.01)	>0.9
NLR	0.176	0.80 (0.54, 1.11)	0.216
PLR	0.006	0.99 (0.98, 1.00)	0.169
SII index (×10^9^/L)	0.001	1	0.311

### 3.3 Nomogram for the improvement of VFD in patients with a pituitary tumor

Four clinical features were selected from the LASSO regression analysis and further stepwise analysis to establish the predictive nomogram, including compression of the optic chiasm, preoperative MD, diffuse defect, and duration of visual symptoms. The nomogram allowed for an estimation of the individual probability of improvement in visual field defects in patients with pituitary tumors (**Figure 2C**). The total number of points was calculated based on the four selected features. By projecting the total points to the lower total points scale, we could predict the probability of improving visual field defects in patients with pituitary tumors. We revealed the contribution of each factor to the improvement of VFD in patients with pituitary tumors. For example, the frequency of improvement in VFD was 33% for pituitary tumor patients with optic chiasm compression, a preoperative MD of 16, a diffuse defect, and a 20-month duration of visual symptoms. Calculations were made as follows: based on the location of each factor in the nomogram, 13 points were given for “optic chiasm compression,” 20 for “preoperative MD 16,” 20 for “diffuse defect,” and 50 for “20-month.” By adding these points together, we obtained 103 points, equivalent to a probability of approximately 33% for the improvement of VFD. Finally,a dynamic nomogram was made. The dynamic nomogram may be obtained at https://cerebralnomogram.shinyapps.io/Pituitary_adenoma_IVFDnom/ .

### 3.4 Validation of a nomogram

**Figure 3:**
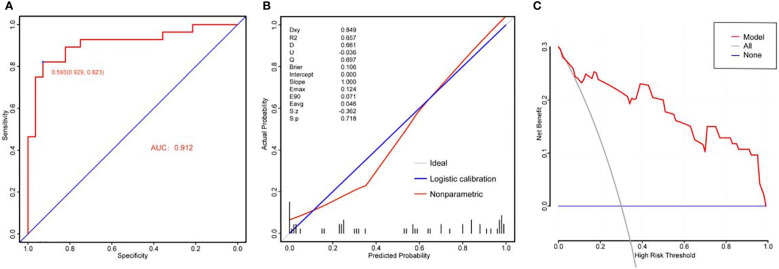
The ROC curves, the calibration curves and the decision curve analysis of the predictive nomogram for the improvement of visual field defect in patients with pituitary tumor. **(A)** The ROC curve of the predictive nomogram, the AUC is 0.912. ROC, receiver operating characteristic; AUC, area under curve; CI, confidence interval. **(B)** The calibration curve of the predictive nomogram; **(C)** The decision curve of the predictive nomogram.

The area under the curve (AUC) of the nomogram was 0.912, indicating that the nomogram had a good degree of differentiation (**Figure 3A**). The constructed calibration curve showed that the nomogram had a good calibration in this study (**Figure 3B**). The calibration curve in our study revealed a good degree of uniformity between the likelihood of predicted improvement and unimproved eyes (Brier = 0.106, S: p = 0.718, Emax = 0.124). Decision curve analysis showed that the nomogram had good clinical application value and could assist in clinical decision-making (**Figure 3C**).

### 3.5 Degrees of improvement in VFD based on different visual ranges

**Figure 4:**
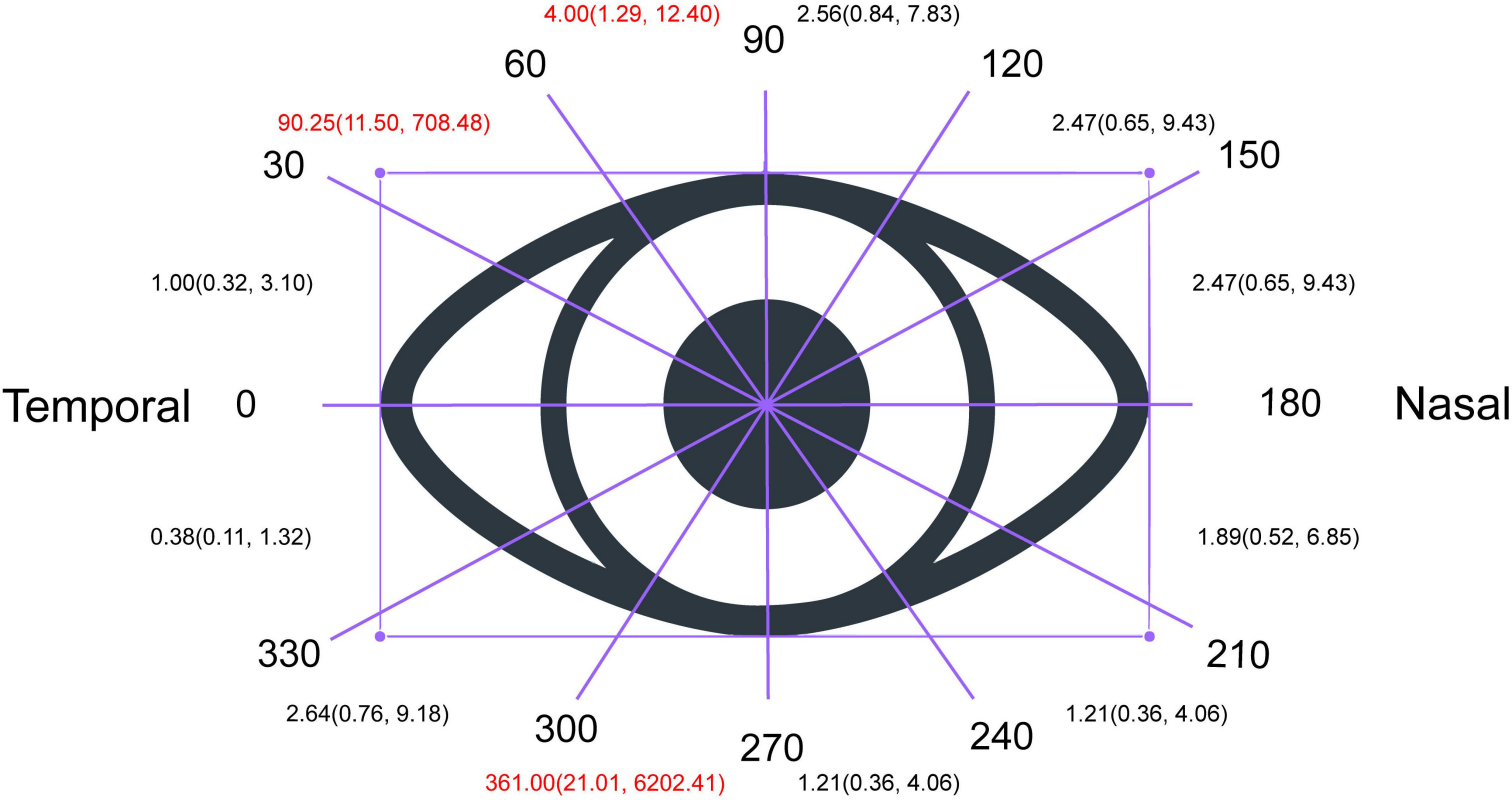
Degree of Improvement of visual field defect based on different visual range. As shown in the supplementary material, we found more improved eyes than unimproved eyes in the overall divided range (RR = 2.48, 95% CI: 1.77–3.48, p < 0.00001). The extent of improvement in eyes with a visual field defect was higher in the range of 30–60° or 270–300° than in other ranges (30–60°: RR = 99.25, 95% CI: 11.50–708.48; 270–300°: RR = 361.00, 95% CI: 21.01–6202.41), which means that a VFD in the range of 270–300° or 30–60° is easier to recover than that in other ranges.

Next, we performed a statistical analysis to determine the degree of improvement of VFD among different visual ranges. As shown in the supplementary material, we found more improved eyes than unimproved eyes in the overall divided range (RR = 2.48, 95% CI: 1.77–3.48, p < 0.00001). The extent of improvement in eyes with a visual field defect was higher in the range of 30–60° or 270–300° than in other ranges (30–60°: RR = 99.25, 95% CI: 11.50–708.48; 270–300°: RR = 361.00, 95% CI: 21.01–6202.41), which means that a VFD in the range of 270–300° or 30–60° is easier to recover than that in other ranges. (**Figure 4**).

### 3.6 Illustrative cases

**Figure 5:**
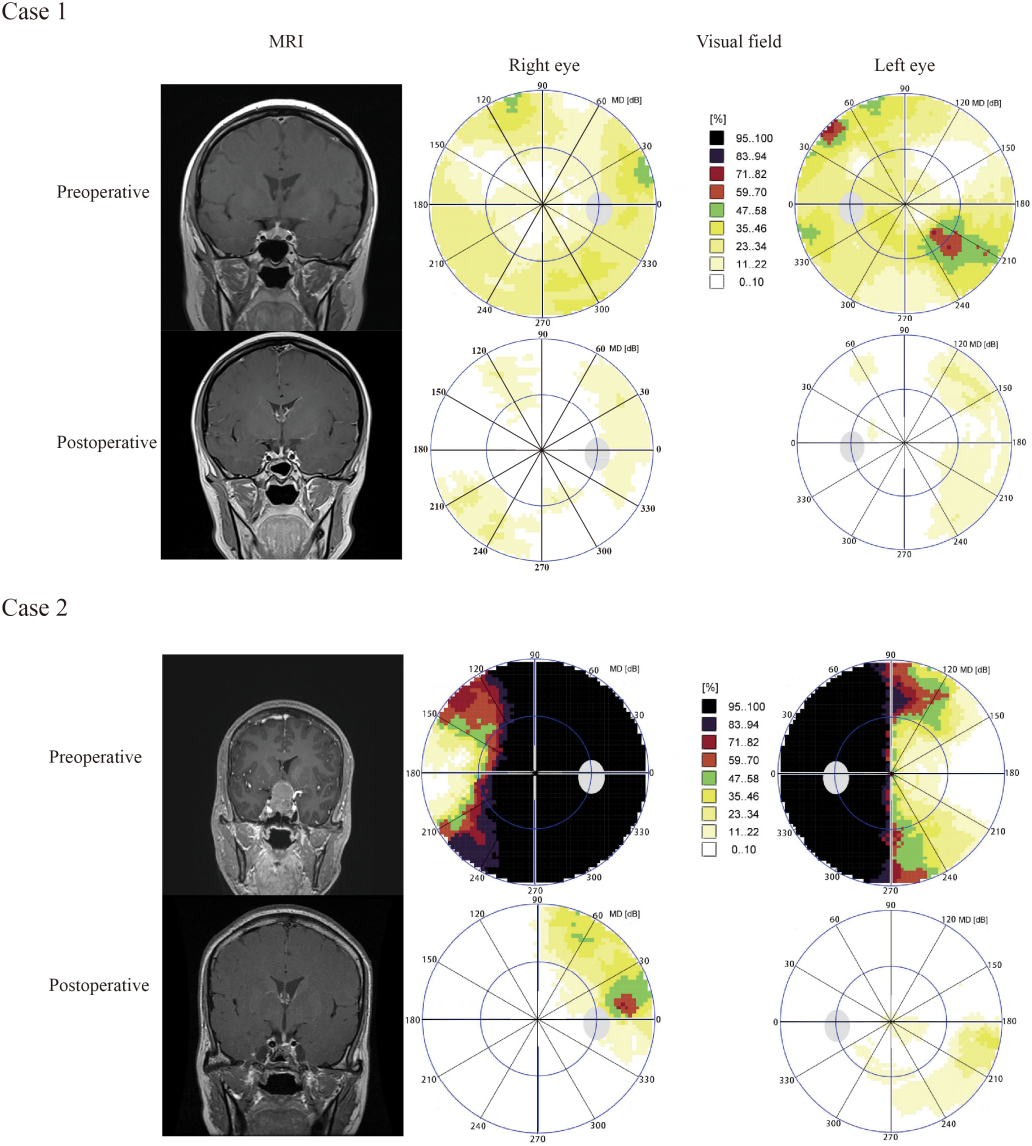
Results of visual field and pituitary MRIs of illustrative cases. Case 1 A 24-year old female was diagnosed with pituitary adenoma and underwent ETSS, who presented with facial acne for 5 months. Preoperative MRI revealed that tumor maximum diameter was 6 millimeters, and the tumor was totally resection identified by postoperative MRI (**Figure 5**, Case 1). Case 2, A 48-year old female with no familial history, who presented with headche and diplopia for 3 months, was admitted to our hosptial. Preoperative MRI suggested that tumor maximum diameter was 35 millimeters, and the tumor was totally resection identified by postoperative MRI (**Figure 5**, Case 2). We chose the endoscopic surgery for her. According to visual field maps (**Figure 5**, Case 2), VF defects were improved in the 270–300°range of inferior temporal quadrant. The VF defect of case 2 were obviously improved than case 1. The patient of case 2 was with compression of the optic chiasm, diffuse defect, higher preoperative mean defect (MD) and duration of the visual symptom, but case 1 not.

Case 1 A 24-year old female was diagnosed with pituitary adenoma and underwent ETSS, who presented with facial acne for 5 months. Preoperative MRI revealed that tumor maximum diameter was 6 millimeters, and the tumor was totally resection identified by postoperative MRI (**Figure 5**, Case 1).

Case 2, A 48-year old female with no familial history, who presented with headche and diplopia for 3 months, was admitted to our hosptial. Preoperative MRI suggested that tumor maximum diameter was 35 millimeters, and the tumor was totally resection identified by postoperative MRI (**Figure 5**, Case 2). We chose the endoscopic surgery for her. According to visual field maps (**Figure 5**, Case 2), VF defects were improved in the 270–300°range of inferior temporal quadrant. The VF defect of case 2 were obviously improved than case 1. The patient of case 2 was with compression of the optic chiasm, heavily diffuse defect, higher preoperative mean defect (MD) andduration of the visual symptom, but case 1 not.

## Discussion

A previous study suggested that VF recovery is associated with many factors, including age, duration of symptoms or a visual impairment, RNFL thickness, tumor volume, suprasellar tumor extension, and expression levels of vascular endothelial growth factor (VEGF)/Ki-67. We developed a predictive model that comprehensively integrates multiple parameters to predict visual recovery after endoscopic surgery in patients with pituitary adenoma. The compression of the optic chiasm, preoperative MD, diffuse defects, and duration of visual symptoms were used to construct the nomogram. The final nomogram showed an AUC of 0.912, indicating good calibration. The inferior temporal region (270–360°) was the most significant recovery region of the VF after ETSS.

VF defects are a common symptom of pituitary adenomas, especially early in the disease’s presentation. Patients with pituitary tumors often visit the ophthalmology department first and not neurosurgery. Patients with pituitary apoplexy require urgent surgical resection, presenting with acute visual deterioration (7). Surgery is an effective treatment for VF defects caused by pituitary adenomas. There are two surgical approaches: endoscopic and microsurgical. In this study, all patients accepted endoscopic transsphenoidal surgery (ETSS).

Several mechanisms underlie VF defects. Direct compression of the optic chiasm can be seen in MRI images of patients with pituitary macroadenomas. This generally results in temporal or bitemporal hemianopia. With disease progression, visual field loss of the nasal pattern can be involved, implying that nasal retinal fibers are preferentially damaged more than the temporal retinal fibers (20). Some scholars have focused on the blood supply system of the optic chiasm. The microcirculation in the median chiasma is weak, causing bitemporal hemianopia. This mechanism also provides evidence that the temporal VF can be preserved due to the lateral chiasma arteries. In addition, although there is no direct compression of the pituitary microadenoma, as demonstrated by MRI, bitemporal hemianopia can be found. “Shunt-flow” (stealing blood) in pituitary microadenomas results in the ischemia of the optic chiasma (21, 22).

In our study, the most significant recovery region of VF after ETSS was in the inferior temporal region (270–360°). A previous study demonstrated that the temporal visual field improved earlier than the nasal visual field and that the inferior temporal quadrant had the greatest relative improvement (23, 24). Improvement in the temporal hemifield was already evident after ETSS (23, 24). The VFD starts from the superior temporal quadrant, which is consistent with the rule that nerve fibers below the optic chiasm are compressed first (20). The postoperative improvement in the visual field was in reverse order of the previous damage, which may be related to the relief of compression after the resection of the pituitary tumor.

The present study demonstrated the correlation of VF recovery with the duration of visual symptoms, optic nerve compression, preoperative MD, and diffuse defects. An inverse correlation was found between symptom duration and postoperative visual field recovery. A previous study suggested that patients with visual symptoms of <1-year duration had better visual outcomes (25). Visual symptoms were caused by compression and ischemia of the optic chiasm. The prolonged visual symptoms may suggest a longer duration of compression and ischemia. This causes irreversible damage to the optic chiasm and visual pathways, which might affect VF recovery (12, 25).

Pituitary macroadenomas mostly affect the visual pathway and cause visual field defects because they compress the optic chiasm. VF recovery mainly depends on defects in the preoperative VF, which indirectly indicates that optic chiasm compression may affect VF recovery (26).Some studies have suggested that preoperative MD negatively correlates with VF recovery (13). Furthermore, evidence supports that a higher MD value of preoperative VF is favorable for VF outcome (27). Our study found that preoperative MD and optic chiasm compression are associated with VF recovery. After tumor resection, compression of the optic chiasm was relieved in patients with a higher preoperative MD. Most patients with obvious preoperative VF defects have good VF outcomes. However, for patients with severe preoperative VFD, the postoperative VF was difficult to improve even after surgical treatment due to irreversible changes caused by long-term compression of the optic chiasm. Otherwise, tumor apoplexy will further aggravate compression of the optic chiasm in the short term, causing severe visual field defects. These patients require early surgical treatment to reduce the risk of irreversible VF impairment. Studies have shown that patients with hemorrhagic pituitary apoplexy require early neurosurgical intervention within 48 hours (28).

Diffuse defects suggest a wide range of VF defects. In this study, most patients with diffuse defects had significant VF recovery after surgical treatment. Although transsphenoidal surgery is an effective treatment, patients with severe preoperative optic chiasm compression or ischemia cannot recover from visual impairment.

Contradictory findings regarding tumor maximum diameter have been reported in the literature. Some studies have shown that tumor maximum diameter can be used as a predictor of postoperative visual outcomes (4, 29). When the tumor maximum diameter is <36.5 mm, better visual field outcomes will be achieved after surgery (25). Among patients with adenomas with a mean diameter size >36.5 mm, postoperative visual field recovery was difficult after ETSS and even after further deterioration. This may be associated with severe compression of the optic chiasm by a macroadenoma, resulting in irreversible mechanical damage to the optic chiasm and the visual pathway. Other studies found no correlation between VF recovery and tumor volume (27). In addition, suprasellar extension (SSE) of the PA is a predictor of visual outcome after ETSS (25, 27). SSE was likely to reflect the severity of optic chiasm compression or ischemia (30). The correlation between VF recovery and tumor maximum diameter should be considered to assess the severity of the preoperative visual impairment.

Few studies have examined the effects of histopathology and immunohistochemistry on visual field recovery. Some studies have suggested that visual field recovery is associated with low expression of VEGF/Ki67 (13). Our study found a significant relationship between VF recovery and low EGFR expression in tumor tissues, but not Ki67 expression. Ki67/EGFR is important for tumor angiogenesis. The relationship between VF recovery and the Ki67/EGFR ratio requires further validation in the field of molecular biology.

In addition, no other significant factors were found in our study, including age, sex, tumor texture, SII index, P53, CD34, and MMP9. There are contradictory results regarding age. One study suggested that a younger age of onset is more likely to be beneficial for visual field deficit improvement (31). The blood supply to the optochiasmatic system is enriched in young patients. Blood flow plays a critical role in VF recovery by nourishing the optic chiasm and the visual pathways. Most elderly patients believe that visual dysfunction is related to presbyopia. They are insensitive to visual impairment and delayed treatment, resulting in the longer optic nerve and optic chiasm compression and a reduced possibility of visual field recovery.

Our study has some limitations. The small number of patients was the main limitation, and the results need to be confirmed in an extended study population. In addition, there were no data on visual acuity for some patients. Despite these limitations, the specific regions and risk factors of VF recovery are not often published in the literature, which is the novelty of this study.

In conclusion, we developed a predictive nomogram model based on factors associated with significant visual field improvement after ETSS in patients with pituitary adenoma. The duration of visual symptoms, optic nerve compression, preoperative MD, and diffuse defects were used as predictive variables. The postoperative improvement in the visual field starts in the range of 270–300° in the inferior temporal quadrant. This would allow tailored counseling for individual patients by precisely predicting the visual field recovery after surgery.

## Data availability statement

The original contributions presented in the study are included in the article/**Supplementary Material**. Further inquiries can be directed to the corresponding authors.

## Ethics statement

The studies involving human participants were reviewed and approved by The First Affiliated Hospital of Soochow University. The patients/participants provided their written informed consent to participate in this study. Written informed consent was obtained from the individual(s) for the publication of any potentially identifiable images or data included in this article.

## Author contributions

XZha and XS contributed to the study conception and design. XJ, XZhu and SY prepared material, analyzed data and illustrated the results. KZ, XL and KY contributed to the data collection and reference collection. XJ wrote the first draft of the manuscript and all authors communicated on previous revisions. All authors approved the final manuscript.

## References

[1] Ostrom QT Gittleman H Truitt G Boscia A Kruchko C Barnholtz-Sloan JS. CBTRUS statistical report: Primary brain and other central nervous system tumors diagnosed in the United States in 2011-2015. Neuro Oncol (2018) 20(suppl_4):iv1–iv86. doi: 10.1093/neuonc/noy131 30445539 PMC6129949

[2] Danesh-Meyer HV Yoon JJ Lawlor M Savino PJ. Visual loss and recovery in chiasmal compression. Prog Retin Eye Res (2019) 73:100765. doi: 10.1016/j.preteyeres.2019.06.001 31202890

[3] Anderson D Faber P Marcovitz S Hardy J Lorenzetti D. Pituitary tumors and the ophthalmologist. Ophthalmology (1983) 90(11):1265–70. doi: 10.1016/s0161-6420(83)34393-1 6664664

[4] Uy B Wilson B Kim WJ Prashant G Bergsneider M. Visual outcomes after pituitary surgery. Neurosurg Clin N Am (2019) 30(4):483–9. doi: 10.1016/j.nec.2019.06.002 31471055

[5] Hedges TR. Preservation of the upper nasal field in the chiasmal syndrome: An anatomic explanation. Trans Am Ophthalmol Soc (1969) 67:131–41. PMC1310336 5381296

[6] Qiao N Ye Z Shou X Wang Y Li S Wang M. Discrepancy between structural and functional visual recovery in patients after trans-sphenoidal pituitary adenoma resection. Clin Neurol Neurosurg (2016) 151:9–17. doi: 10.1016/j.clineuro.2016.09.005 27728836

[7] Galal A Ahmed OEF. Determinants of visual and endocrinological outcome after early endoscopic endonasal surgery for pituitary apoplexy. Surg Neurol Int (2022) 23:13:433. doi: 10.25259/SNI_642_2022 PMC9609952 36324938

[8] Sriram PR Sellamuthu P Ghani ARI. Factors affecting visual field outcome post-surgery in sellar region tumors: Retrospective study. Malays J Med Sci (2017) 24(6):58–67. doi: 10.21315/mjms2017.24.6.7 29379387 PMC5771516

[9] Chung YS Na M Yoo J Kim W Jung IH Moon JH. Optical coherent tomography predicts long-term visual outcome of pituitary adenoma surgery: New perspectives from a 5-year follow-up study. Neurosurgery (2020) 88(1):106–12. doi: 10.1093/neuros/nyaa318 32735666

[10] Muskens IS Zamanipoor Najafabadi AH Briceno V Lamba N Senders JT van Furth WR. Visual outcomes after endoscopic endonasal pituitary adenoma resection: a systematic review and meta-analysis. Pituitary (2017) 20(5):539–52. doi: 10.1007/s11102-017-0815-9 PMC5606952 28643208

[11] Shen M Chen Z Shou X Ma Z Ye Z He W. Surgical outcomes and predictors of visual function alterations after transcranial surgery for Large-to-Giant pituitary adenomas. World Neurosurg (2020) 141:e60–9. doi: 10.1016/j.wneu.2020.04.151 32353541

[12] Dhasmana R Nagpal RC Sharma R Bansal KK Bahadur H. Visual fields at presentation and after trans-sphenoidal resection of pituitary adenomas. J Ophthalmic Vis Res (2011) 6(3):187–91. PMC3306097 22454734

[13] Yu FF Chen LL Su YH Huo LH Lin XX Liao RD. Factors influencing improvement of visual field after trans-sphenoidal resection of pituitary macroadenomas: a retrospective cohort study. Int J Ophthalmol (2015) 8(6):1224–8. doi: 10.3980/j.issn.2222-3959.2015.06.27 PMC4651894 26682178

[14] Lee J Kim SW Kim DW Shin JY Choi M Oh MC. Predictive model for recovery of visual field after surgery of pituitary adenoma. J Neurooncol (2016) 130(1):155–64. doi: 10.1007/s11060-016-2227-5 27476080

[15] van Essen MJ Muskens IS Lamba N Belunek SFJ van der Boog ATJ Amelink GJ. Visual outcomes after endoscopic endonasal transsphenoidal resection of pituitary adenomas: Our institutional experience. J Neurol Surg B Skull Base (2021) 82(Suppl 3):e79–87. doi: 10.1055/s-0039-3402020 PMC8289550 34306920

[16] Pelsma ICM Verstegen MJT de Vries F Notting IC Broekman MLD van Furth WR. Quality of care evaluation in non-functioning pituitary adenoma with chiasm compression: visual outcomes and timing of intervention clinical recommendations based on a systematic literature review and cohort study. Pituitary (2020) 23(4):417–29. doi: 10.1007/s11102-020-01044-0 PMC7316692 32419072

[17] Holló G. Comparison of structure-function relationship between corresponding retinal nerve fibre layer thickness and octopus visual field cluster defect values determined by normal and tendency-oriented strategies. Br J Ophthalmol (2017) 101(2):150–4. doi: 10.1136/bjophthalmol-2015-307759 27107030

[18] Mahmoud OM Tominaga A Amatya VJ Ohtaki M Sugiyama K Sakoguchi T. Role of PROPELLER diffusion-weighted imaging and apparent diffusion coefficient in the evaluation of pituitary adenomas. Eur J Radiol (2011) 80(2):412–7. doi: 10.1016/j.ejrad.2010.05.023 20580505

[19] McEligot AJ Poynor V Sharma R Panangadan A. Logistic LASSO regression for dietary intakes and breast cancer. Nutrients (2020) 31 12(9):2652. doi: 10.3390/nu12092652 PMC7551912 32878103

[20] Ogra S Nichols AD Stylli S Kaye AH Savino PJ Danesh-Meyer HV. Visual acuity and pattern of visual field loss at presentation in pituitary adenoma. J Clin Neurosci (2014) 21(5):735–40. doi: 10.1016/j.jocn.2014.01.005 24656736

[21] Lao Y Gao H Zhong Y. Vascular architecture of the human optic chiasma and bitemporal hemianopia. Chin Med Sci J (1994) 9(1):38–44. doi: 10.1076/orbi.22.2.81.14316 8086633

[22] van Overbeeke J Sekhar L. Microanatomy of the blood supply to the optic nerve. Orbit (2003) 22(2):81–8. doi: 10.1076/orbi.22.2.81.14316 12789588

[23] Kerrison JB Lynn MJ Baer CA Newman SA Biousse V Newman NJ. Stages of improvement in visual fields after pituitary tumor resection. Am J Ophthalmol (2000) 130(6):813–20. doi: 10.1016/s0002-9394(00)00539-0 11124302

[24] Jakobsson KE Petruson B Lindblom B. Dynamics of visual improvement following chiasmal decompression. quantitative pre- and postoperative observations. Acta Ophthalmol Scand (2002) 80(5):512–6. doi: 10.1034/j.1600-0420.2002.800510.x 12390163

[25] Thotakura AK Patibandla MR Panigrahi MK Addagada GC. Predictors of visual outcome with transsphenoidal excision of pituitary adenomas having suprasellar extension: A prospective series of 100 cases and brief review of the literature. Asian J Neurosurg (2017) 12(1):1–5. doi: 10.4103/1793-5482.149995 28413523 PMC5379778

[26] Gnanalingham KK Bhattacharjee S Pennington R Ng J Mendoza N. The time course of visual field recovery following transphenoidal surgery for pituitary adenomas: predictive factors for a good outcome. J Neurol Neurosurg Psychiatry (2005) 76(3):415–9. doi: 10.1136/jnnp.2004.035576 PMC1739567 15716538

[27] Park SH Kang MS Kim SY Lee JE Shin JH Choi H. Analysis of factors affecting visual field recovery following surgery for pituitary adenoma. Int Ophthalmol (2021) 41(6):2019–26. doi: 10.1007/s10792-021-01757-6 33625650

[28] Seuk JW Kim CH Yang MS Cheong JH Kim JM. Visual outcome after transsphenoidal surgery in patients with pituitary apoplexy. J Korean Neurosurg Soc (2011) 49(6):339–44. doi: 10.3340/jkns.2011.49.6.339 PMC3158476 21887391

[29] Jeon C Park KA Hong SD Choi JW Seol HJ Nam DH. Clinical efficacy of optical coherence tomography to predict the visual outcome after endoscopic endonasal surgery for suprasellar tumors. World Neurosurg (2019) 132:e722–e31. doi: 10.1016/j.wneu.2019.08.031 31421301

[30] Luomaranta T Raappana A Saarela V Liinamaa MJ. Factors affecting the visual outcome of pituitary adenoma patients treated with endoscopic transsphenoidal surgery. World Neurosurg (2017) 105:422–31. doi: 10.1016/j.wneu.2017.05.144 28583452

[31] Barzaghi LR Medone M Losa M Bianchi S Giovanelli M Mortini P. Prognostic factors of visual field improvement after trans-sphenoidal approach for pituitary macroadenomas: review of the literature and analysis by quantitative method. Neurosurg Rev (2012) 35(3):369–78. doi: 10.1007/s10143-011-0365-y. discussion 78-9. 22080165

