# Supplementary figures and images for: Visual field improvement after endoscopic transsphenoidal surgery in patients with pituitary adenoma
Source: Front Oncol. 2023 Feb 15;13:1108883. doi: 10.3389/fonc.2023.1108883 (PMC9975539; doi:10.3389/fonc.2023.1108883)

## Analysis for visual field defects among different visual ranges

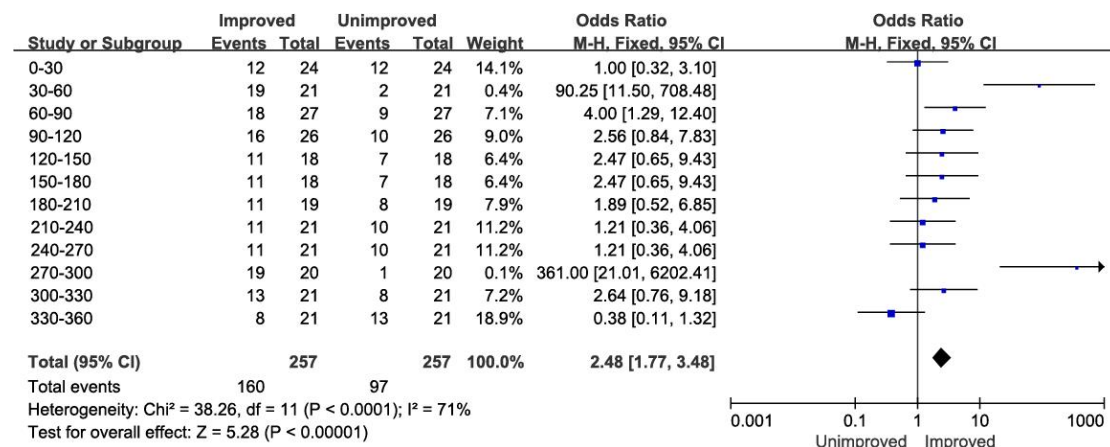

Supplement: Supplementary file 1 [file DataSheet_1.pdf]
